# A new opportunity for MEMRI

**DOI:** 10.18632/aging.101283

**Published:** 2017-08-28

**Authors:** Ryan Cloyd, Moriel Vandsburger, Jose F. Abisambra

**Affiliations:** Sanders-Brown Center on Aging and Department of Physiology, College of Medicine, University of Kentucky, Lexington, KY 40536-0230, USA

**Keywords:** MEMRI, neurodegeneration, manganese, MnDPDP

Manganese enhanced magnetic resonance imaging (MEMRI) has long been recognized as a potential tool for radiologic studies. Lin and Koretsky significantly expanded the utility of MEMRI when they showed that it could provide non-invasive, *in vivo* measurement of neuronal activity [1]. We recently showed that MEMRI with DR1 mapping detects significant differences in neuronal function between groups of wild type and tau transgenic mice [2]. This study reinforces the potential of MEMRI to be used in the early detection of neuronal dysfunction associated with neurodegeneration by showing differences in function before the development of significant cognitive changes. A major challenge in developing effective treatments for neurodegenerative conditions is identifying patients while still within a window of time where pharmacologic intervention can be effective. Many drugs have shown disease-modifying potential in animal models, but all have failed in clinical trials. Therefore, MEMRI has the potential to contribute to solving this problem, and implementation of the technique in human populations may allow more effective recruiting of participants for drug trials.

A major hurdle translating MEMRI to humans is the concern for toxicity upon overexposure to manganese that may be associated with isolated or repeated administration. Much of this concern stems from the MSDS for manganese chloride, the most commonly used manganese agent. However, MEMRI studies have often exceeded the listed toxic doses without ill effects [3]. Despite this and many additional examples demonstrating the safety of manganese administration at doses required for imaging, concern remains regarding MEMRI in patients. In response, development of mangafodipir (Teslascan, MnDPDP), a FDA approved contrast agent, became a viable option to assuage these concerns. Many clinical trials were performed using MnDPDP in human populations in the early 1990s, finding headache and nausea as the most severe side effects reported by patients [4]. Most individuals exposed to MnDPDP experience only facial flushing that subsides within minutes of administration.

While numerous pre-clinical studies demonstrated toxicity of MnCl_2_ at high doses, MnDPDP exhibits no major toxicity in a variety of animal models at doses much higher than those required for adequate image enhancement [5]. Moreover, multiple clinical trials demonstrated tolerability to MnDPDP in human subjects. These data suggest that with further safety evaluation, MnDPDP may enable comprehensive MEMRI examination in humans.

Although it remains a FDA-approved drug, MnDPDP is not widely used for imaging studies. Currently, the therapeutic role of MnDPDP is being investigated including as a chemotherapy adjunct to prevent drug-induced neuropathy and as a cardio-protectant following acute myocardial infarction. The fodipir ligand component of MnDPDP is a structural derivative of vitamin B6 (which is commonly used to treat certain types of neuropathy), but the anti-neuropathic effects of MnDPDP more likely stem from its ability to scavenge superoxide anions generated by many chemo-therapeutics than from this structural similarity [6]. The superoxide dismutase (SOD) activity of MnDPDP is likely also the basis of its ability to prevent reperfusion observed in a small study of human patients with acute ST-elevation myocardial infarction [7]. Studies are ongoing, but MnDPDP can be expected to attain a more significant therapeutic role as its properties and activity within the body are better understood. Therapeutic doses generally exceed those required for imaging, so advances in therapeutic studies, particularly those demonstrating tolerability, will help pave the way for future imaging applications.

Importantly, advances in the preclinical application of MEMRI occurred concomitantly with technological advances in imaging including high field 7T human brain imaging, combined positron emission tomography (PET) and MRI imaging, and targeted PET tracers for the study of neurodegenerative diseases. While early studies of MnDPDP that utilized clinical imaging systems were restricted to field strengths of 1.5T and 3T, the increasing adoption of 7T human brain imaging offers several important advantages. Beyond the ability to resolve smaller substructures in the brain, the longer T1 relaxation times of brain tissues at 7T extends the dynamic range for quantitation of changes in T1 due to manganese accumulation. Subsequently, further reductions in MnDPDP dose could be pursued without attenuation of sensitivity. Alternatively, existing doses could be maintained and more subtle changes in accumulation could be quantified. Finally, targeted PET agent studies can be performed in parallel to MEMRI, allowing for multiplexed molecular imaging of neurodegenerative disease biomarkers and neuronal function. Together, these could provide a powerful toolbox for comprehensive and earlier detection, as well as longitudinal monitoring of response to pharma-ceutical interventions that seek to attenuate disease progression.

Therefore, MnDPDP has further use as an agent for MEMRI. Despite safety concerns, numerous studies [4][7]) show that MnDPDP is well tolerated in human populations at the doses required for imaging or therapeutic benefit. We are currently exploring a role for MnDPDP in the imaging paradigm described in [2], with promising early results. Once the efficacy of MnDPDP to detect changes in neuronal function has been established, the door will open for translational studies involving human patients. MnDPDP was quickly supplanted in its original role for imaging, but recent years have given it a new purpose. The near future may see a major return of mangafodipir to the market and opportunities for MEMRI in translational research.
